# Integrated Metagenomic and Metabolomics Profiling Reveals Key Gut Microbiota and Metabolites Associated with Weaning Stress in Piglets

**DOI:** 10.3390/genes15080970

**Published:** 2024-07-23

**Authors:** Xianrui Zheng, Liming Xu, Qingqing Tang, Kunpeng Shi, Ziyang Wang, Lisha Shi, Yueyun Ding, Zongjun Yin, Xiaodong Zhang

**Affiliations:** 1 College of Animal Science and Technology, Anhui Agricultural University, Hefei 230036, China; ahauzxr@ahau.edu.cn (X.Z.); 2022205006@stu.njau.edu.cn (L.X.); tangqingqing@stu.ahau.edu.cn (Q.T.); 21720410@stu.ahau.edu.cn (K.S.); wangziyang@stu.ahau.edu.cn (Z.W.); shilisha@stu.ahau.edu.cn (L.S.); dingyueyun@ahau.edu.cn (Y.D.); 2 Key Laboratory of Local Animal Genetic Resources Conversion and Bio-Breeding of Anhui Province, Hefei 230036, China

**Keywords:** weaning stress, piglet, gut microbiota, metagenomic, LC–MS/MS-based metabolomics

## Abstract

(1) Background: Weaning is a challenging and stressful event in the pig’s life, which disrupts physiological balance and induces oxidative stress. Microbiota play a significant role during the weaning process in piglets. Therefore, this study aimed to investigate key gut microbiota and metabolites associated with weaning stress in piglets. (2) Methods: A total of ten newborn piglet littermates were randomly assigned to two groups: S (suckling normally) and W (weaned at 21 d; all euthanized at 23 d). Specimens of the cecum were dehydrated with ethanol, cleared with xylene, embedded in paraffin, and cut into 4 mm thick serial sections. After deparaffinization, the sections were stained with hematoxylin and eosin (H&E) for morphometric analysis. Cecal metagenomic and liver LC-MS-based metabolomics were employed in this study. Statistical comparisons were performed by a two-tailed Student’s *t*-test, and *p* < 0.05 indicated statistical significance. (3) Results: The results showed that weaning led to intestinal morphological damage in piglets. The intestinal villi of suckling piglets were intact, closely arranged in an orderly manner, and finger-shaped, with clear contours of columnar epithelial cells. In contrast, the intestines of weaned piglets showed villous atrophy and shedding, as well as mucosal bleeding. Metagenomics and metabolomics analyses showed significant differences in composition and function between suckling and weaned piglets. The W piglets showed a decrease and increase in the relative abundance of *Bacteroidetes* and *Proteobacteria* (*p* < 0.05), respectively. The core cecal flora in W piglets were *Campylobacter* and *Clostridium*, while those in S piglets were *Prevotella* and *Lactobacillus*. At the phylum level, the relative abundance of *Bacteroidetes* significantly decreased (*p* < 0.05) in weaned piglets, while *Proteobacteria* significantly increased (*p* < 0.05). Significant inter-group differences were observed in pathways and glycoside hydrolases in databases, such as the KEGG and CAZymes, including fructose and mannose metabolism, salmonella infection, antifolate resistance, GH135, GH16, GH32, and GH84. We identified 757 differential metabolites between the groups through metabolomic analyses—350 upregulated and 407 downregulated (screened in positive ion mode). In negative ion mode, 541 differential metabolites were identified, with 270 upregulated and 271 downregulated. Major differential metabolites included glycerophospholipids, histidine, nitrogen metabolism, glycine, serine, threonine, β-alanine, and primary bile acid biosynthesis. The significant differences in glycine, serine, and threonine metabolites may be potentially related to dysbiosis caused by weaning stress. Taken together, the identification of microbiome and metabolome signatures of suckling and weaned piglets has paved the way for developing health-promoting nutritional strategies, focusing on enhancing bacterial metabolite production in early life stages.

## 1. Introduction

Weaning is a crucial driver of pig productivity and pig farm profits. Early weaning shortens the pig slaughter cycle and controls the spread of disease, which can improve sow productivity. However, early weaning is accompanied by the cessation of suckling and a change in diet and environment, leading to physiological and psychological stress in piglets [1,2]. Early weaning can impair the gastrointestinal structure and function of piglets and promote gastrointestinal disorders, including diarrhea, growth obstruction, impaired immune function, and increased intestinal tissue permeability and inflammation [3]. During early life, microbial abundance may vary between piglets and is sensitive to the influence of various factors. Gut microbial community structure in newborn piglets is collectively affected by the initial maternal environment, early diet, and external stimuli. With age, the intestinal microbial structure changes into a mature and stable state [4]. To promote farm productivity and animal health, it is important to understand the effects of early weaning stress on the structure and function of intestinal flora and hepatic metabolites.

The mammalian intestine houses trillions of microbes that participate in maintaining host health/inhibiting gastrointestinal disorders. Growing evidence suggests that the targeted reconstitution of gut microbiota can counteract gastrointestinal disorders. A reduction in normal flora contents limits the protection of the intestinal tract, promotes the proliferation of pathogenic bacteria, and promotes broader dysbiosis in piglets. Enterococci and Escherichia coli are the core risk flora for diarrhea in newborn piglets [5]. *Fusobacterium* may also cause diarrheal enteritis [6]. Normal microbial structure is integral for maintaining normal carbohydrate digestion and fermentation, producing short-chain fatty acids, maintaining intestinal villi function, regulating immune response, and inhibiting pathogen growth. As the stress center of the body, gut dysfunctions may lead to wider organ dysfunctions. The dysbiosis of intestinal microbiota may be a key driver of weaning stress. Short-chain fatty acid (SCFA) and metabolite contents have also been shown to participate in the regulation of intestinal tissue development, immune function, and host metabolism [7]. Weaning is the most stressful time in a piglet’s life, especially because of the abrupt switch from milk to solid diets. Accumulating evidence indicates that piglets simultaneously experience an abrupt shift in gut microbiota structure and function at this time [8,9,10], leading to poor growth performance or even death [11]. However, the changes in gut microbial composition induced by weaning stress in piglets remain largely unclear.

Numerous recent studies have investigated the influence of gut microbiota and metabolites on host physiology and organ function [12,13,14,15,16]. The liver is an essential metabolic organ that is intimately coupled to the intestinal environment via the portal venous system [12,17]. The gut–liver axis participates in various biological processes [18]. To determine the broader role of gut microbiota under weaning stress, it is important to characterize the metabolomic changes in liver markers. This could also reveal the drivers of microbial structure and mechanisms underlying their beneficial effects. 

In this study, a total of ten sibling piglets, [Duroc × (Landrace × Yorkshire)] were randomly allotted into two groups with five replicate pens: suckling normally (S) and weaned at 21 d (W). High-throughput metagenomic sequencing was utilized to screen core flora and determine the microbial community structure and function in the cecum of S and W piglets. We further analyzed the changes in the liver metabolic markers of S and W piglets using high-resolution liquid chromatography and mass spectrometry. Our study elaborates on the mechanisms of stress in weaned piglets and provides a theoretical basis for developing more effective management strategies.

## 2. Materials and Methods

### 2.1. Ethics Statement

All procedures involving animal experiments were conducted in accordance with the guidelines for the care and use of experimental animals established by the Institutional Animal Care and Use Committee of Anhui Agricultural University, Hefei, China, under permit number AHAU 20190115.

### 2.2. Study Design and Animals

A total of 10 sibling piglets (Duroc × (Landrace × Yorkshire)) with similar initial body weights were selected and exclusively sow reared. No antibiotic therapy was administered. No previous history of viral infections was found on the farm, including PRRSV, PCV2, or diarrhea-related viruses. The piglets were randomly divided into two groups on day 21 after birth: five were weaned at 21 d (W) and another five suckled normally (S). At the age of 21 days, the W piglets were separated from the sows and had ad libitum access to water and feed (*n* = 5/group). After weaning, 10 piglets were subjected to anterior vena cava blood collection using a 5 mL non-anticoagulant tube at 3 d post-weaning. The samples were then stored at 4 °C. At the age of 24 days, all piglets were euthanized. 

After euthanasia by exsanguination from the neck with 75% alcohol disinfection, the abdomens were opened with a sterile surgical blade, cecum segments were isolated and tied off with alcohol-soaked thread to prevent the leakage of intestinal contents, and then immersed in PBS and placed in 15 mL centrifuge tubes containing a 4% paraformaldehyde fixation solution for preservation at 4 °C. Cecal content was collected and placed in 2 mL cryovials, subsequently stored in liquid nitrogen at –80 °C and transported back to the laboratory. Due to operational errors during transportation, one sample in group S was contaminated and only nine samples were used for the following analysis. Specimens of the cecum were dehydrated with ethanol, cleared with xylene, embedded in paraffin, and cut into 4 mm thick serial sections. After deparaffinization, the sections were stained with hematoxylin and eosin (H&E) for morphometric analysis.

### 2.3. Serum Parameter Analysis

Naturally precipitated serum was collected and kept at −20 °C. The serum concentrations of diamine oxidase (DAO), endotoxin (ET), diamine oxidase cortisol, and noradrenaline (NE) were determined using a commercial kit (Shanghai Jianglai Bioengineering Institute, Shanghai, China) according to the manufacturer’s instructions.

### 2.4. Metagenomic Sequencing

Total microbial genomic DNA of cecum contents was extracted using a TIANamp Stool DNAKit (Cat. No. DP328, TIANGEN, Beijing, China), following the instructions provided by the manufacturer. We detected the purity and quality of the genomic DNA using 1% agarose gel and a Nanodrop 2000 UV-VIS spectrophotometer (Thermo Fisher Scientific, Wilmington, NC, USA). DNA samples meeting the quality standards were randomly interrupted into approximately 350 bp fragments with a Covaris ultrasonic crusher. The qualified DNA was amplified, and the metagenome sequencing library was prepared using the TruSeq DNA Sample Prep Kit (Illumina, San Diego, CA, USA). Library quality was measured by qPCR (library effective concentration > 3 nM). Further, metagenome sequencing was conducted on an Illumina HiSeq PE150 platform (Illumina). 

### 2.5. Metagenomic Data Analysis

The raw data sequence reads were trimmed using Trimmomatic (http://www.usadellab.org/cms/?page=trimmomatic, accessed on 22 January 2021). Subsequently, assembly was conducted using Megahit (version v1.0.6, https://github.com/voutcn/megahit, accessed on 15 October 2019), with parameter settings identical to those used for single-sample assembly. The scaffolds from the mixed assembly were also broken at the N connections to yield scaffolds. Both the single-sample and mixed-assembly generated scaffolds were filtered to remove fragments shorter than 500 bp and were then subjected to statistical analysis. We utilized the MetaGeneMark-2 tool (https://github.com/gatech-genemark/MetaGeneMark-2, accessed on 7 April 2021) to predict open reading frames (ORFs) in the scaffolds (≥500 bp) from individual samples and mixed assemblies. Predictions shorter than 100 nucleotides were filtered out, and default parameters were applied for the ORF prediction. The predicted ORFs were then subjected to redundancy removal using CD-HIT software (v4.5.8, http://www.bioinformatics.org/cd-hit/, accessed on 1 September 2009) to generate a non-redundant initial gene catalog, with parameter settings of -c 0.95, -G 0, -aS 0.9, -g 1, and -d 0. Bowtie2 (version 2.2.4) was employed to map the clean data from each sample against the initial gene catalog, calculating the number of reads aligning with the genes. Parameters were set to --end-to-end, --sensitive, -I 200, and -X 400. Genes with read counts ≤2 in any sample were filtered out, resulting in a final gene catalog (Unigenes) for further analysis. The abundance of each gene in the samples was calculated based on the number of aligned reads and gene length. DIAMOND software (v2.1.9, https://github.com/bbuchfink/diamond/, accessed on 31 January 2024) was used to align the Unigenes against the functional databases, KEGG and CAZy. For each sequence alignment result, the Best Blast Hit was selected for subsequent analysis. The relative abundance across various functional levels was tallied, and starting from the abundance tables of each taxonomic level, the number of annotated genes was counted and an overview of relative abundance was presented. Abundance clustering heatmaps were displayed, along with PCA and NMDS dimensionality reduction analyses. Anosim based on functional abundance was conducted for inter-group difference analysis and comparative analysis of metabolic pathways, and inter-group functional difference analyses using Metastat and LEfSe methods were performed.

### 2.6. Metabolomic Profiling 

First, 50 mg of liver tissue was homogenized with 1000 μL of an extraction solution (methanol/acetonitrile/water = 2:2:1, containing an isotopically labeled internal standard mixture) at 35 Hz for 4 min, followed by 5 min of sonication in an ice bath. The homogenization and sonication process was repeated three times. The samples were incubated at 40 °C for 1 h and then centrifuged at 4 °C for 15 min at 12,000 rpm. The supernatant was carefully transferred to a fresh glass vial for analysis. 

LC–MS/MS was performed using a UHPLC system (Vanquish, Thermo Fisher Scientific) with a UPLC BEH Amide column (2.1 mm × 100 mm, 1.7 μm) coupled to a Q Exactive HF-X mass spectrometer (Orbitrap MS, Thermo Fisher Scientific). The mobile phase consisted of 25 mmol/L ammonium acetate and 25 mmol/L ammonia hydroxide in water (pH = 9.75, A) and acetonitrile (B). The auto-sampler temperature and injection volume were set to 4 °C and 3 μL, respectively. 

The Q Exactive HF-X mass spectrometer was used to acquire MS/MS spectra in the information-dependent acquisition (IDA) mode using Xcalibur software (v0.1.4, https://github.com/nickdelgrosso/XCaliburMethodReader, accessed on 29 December 2018). In this mode, the acquisition software continuously evaluates the full scan MS spectrum. The system was operated in positive/negative polarity mode with a spray voltage of 3.2 kV, a capillary temperature of 320 °C, a sheath gas flow rate of 40 arb, and an aux gas flow rate of 10 arb. 

### 2.7. Metabolomics Data Analysis

Raw data were converted into mzXML format using the ProteoWizard software (https://github.com/ProteoWizard/pwiz, accessed on 5 January 2022), followed by peak identification, extraction, alignment, and integration processes performed with R packages. Subsequently, these data were matched against the in-house secondary mass spectrometry database BiotreeDB (version 2.1) for substance annotation, with an algorithm score cutoff set at 0.3. Principal Component Analysis (PCA) and Partial Least Squares Discriminant Analysis (PLS-DA) were conducted using R software (v4.1.3) packages and SIMCA-P 14 (Umetrics, Sweden). Differential metabolites were screened based on criteria including Variable Importance in the Projection (VIP) values > 1, fold changes (FCs) > 2 or <0.5, and *p*-values < 0.05. The online tool MetaboAnalyst was employed for the enrichment analysis of differential metabolites in metabolic pathways, with selection criteria of the Impact Factor (IF) > 0.1 and *p*-values < 0.05. The Kyoto Encyclopedia of Genes and Genomes (KEGG) database was used to identify the pathways associated with the differential metabolites (https://www.genome.jp/KEGG/pathway.html, accessed on 1 July 2024). 

### 2.8. Statistical Analysis

We conducted statistical analyses on serum parameters, cecal microbial taxa (phylum and genus), KEGG pathways, CAZymes, and liver metabolites using the Mann–Whitney test in SPSS software version 26.0 (IBM Corporation, Armonk, NY, USA). Differences were deemed significant if *p* ≤ 0.05. To enhance rigor, the resulting *p*-values underwent false discovery rate (FDR) correction, with statistical significance declared at adjusted *p* ≤ 0.05.

Spearman correlation analysis between the microbial species affected and metabolites was performed using the Spearman correlation test within the R programming environment. Specifically, correlations with an absolute correlation coefficient (|r|) greater than 0.65 and a *p*-value of less than 0.05 were regarded as statistically significant and used to construct a correlation network graph. 

## 3. Results

### 3.1. Differences of Morphological and Plasma Physiological between Weaning and Suckling Piglets

**Figure 1:**
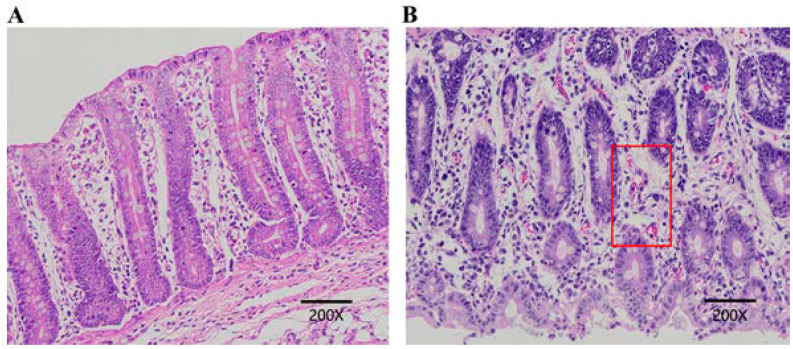
Morphological analysis of HE-stained cecum sections. (**A**) Suckling piglets (S), (**B**) weaned piglets (W), the red box indicates an area of mucosal bleeding (scale bar: 200 µm).

We investigated the effect of weaning stress on the cecum structure of weaned and suckling piglets using hematoxylin–eosin (HE) staining and light microscopy (200× magnification). Major differences were found between W and S piglets. In S piglets, the intestinal villi structure was intact, arranged tightly and orderly, and had a finger-like shape; the columnar epithelial cells had clear outlines (Figure 1A). In contrast, the intestinal tissues of W piglets showed obvious villi atrophy and shedding; bleeding was observed in the mucous membrane (red box in Figure 1B). 

**Figure 2:**
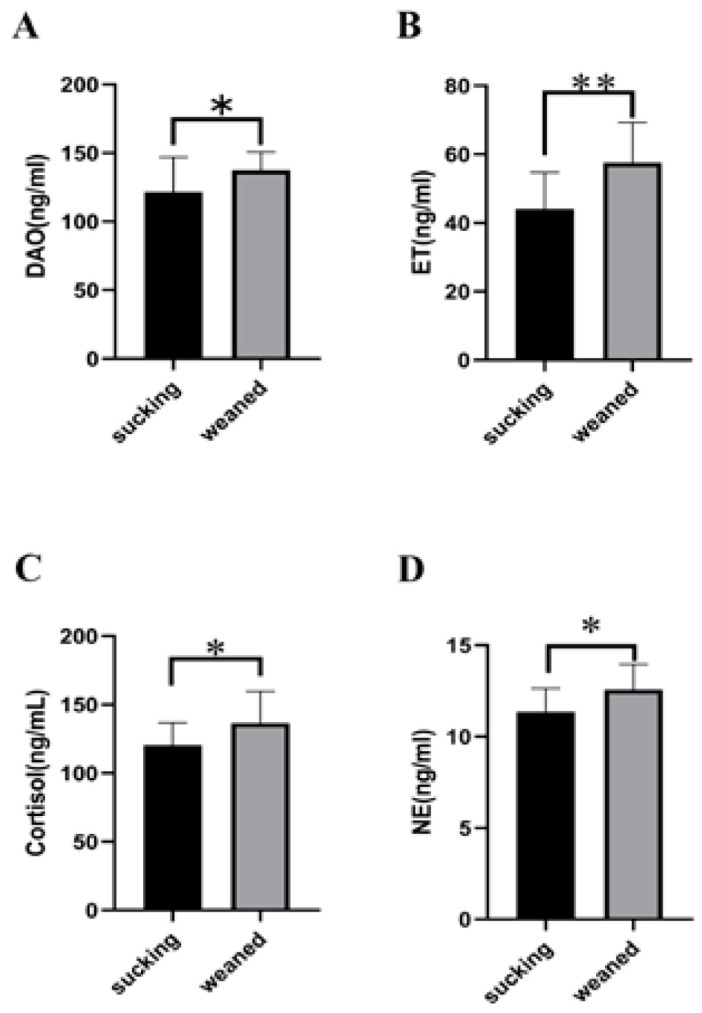
Concentrations of serum parameters in suckling and weaned piglets: (**A**) DAO, (**B**) ET, (**C**) cortisol, (**D**) NE. * indicates *p* < 0.05 and ** indicates *p* < 0.01.

We evaluated the effects of weaning stress on the concentrations of serum parameters and found that serum ET, DAO (Figure 2B, Table S1), cortisol (Figure 2C), and NE (Figure 2D) were higher in weaned than in suckling piglets (*p* < 0.05). These results indicated that weaning stress disturbed the structure of intestinal microflora.

### 3.2. Quality Assessment of Metagenomic Sequencing Data

A total of nine sibling piglets (Duroc × (Landrace × Yorkshire) were subjected to metagenome sequencing. After quality control, a total of 58.26 Gb of clean data were obtained, with an average sequencing depth of 6.47 Gb for each sample. These data showed that the depth and effective data rate of our sequencing met the requirements of subsequent analysis.

### 3.3. Differences of Gut Microbial Diversity and Composition in Piglets between the Two Groups

**Figure 3:**
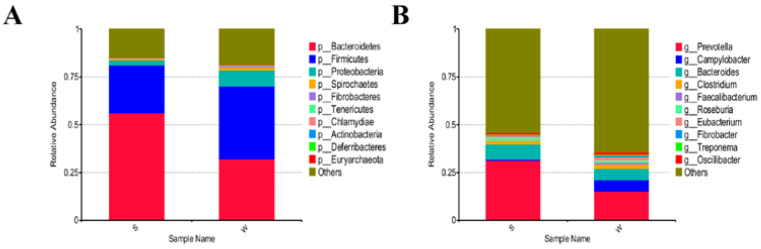
Relative abundances (%) of core phyla (**A**) and genera (**B**). Bars S and W represent the relative abundances in suckling and weaned piglets, respectively.

We explored the effects of weaning stress on intestinal microbiome structure through metagenomic sequencing of cecum samples. The top three dominant bacterial phyla in both suckling and weaned piglets were *Bacteroidetes*, *Firmicutes*, and *Proteobacteria*, accounting for 56.19%, 24.88%, and 2.61%, respectively, of bacteria in S piglets and 32.01%, 38.17%, and 8.46%, respectively, of bacteria in W piglets (Figure 3A). The top three genera in suckling piglets were *Prevotella*, *Bacteroides*, and *Clostridium*, accounting for 31.36%, 7.82%, and 2.03%, respectively, of all bacteria. The top three genera in weaned piglets were *Prevotella*, *Campylobacter*, and *Bacteroides*, accounting for 15.20%, 5.97%, and 5.84%, respectively, of bacteria (Figure 3B). 

**Figure 4:**
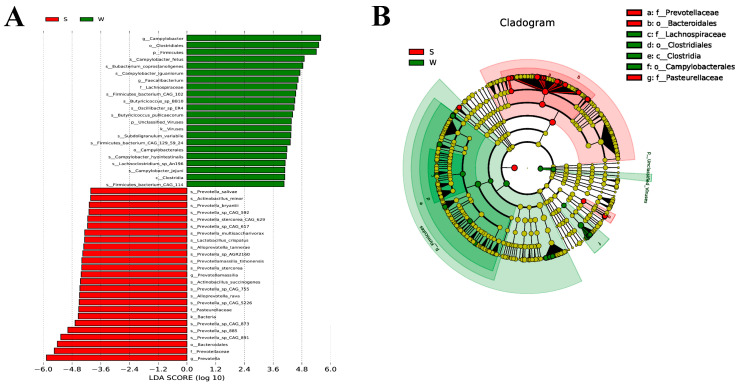
LEfSe analysis. (**A**) LDA score showing the differential biomarkers. The length of the column represents the LDA score. (**B**) A cladogram of relationships between taxa; phyla and genera are presented from the inside out. Each small circle at different classification levels represents a taxon (the diameter of the circle is proportional to the relative abundance). The shared/common species are colored yellow; biomarkers are colored based on group. S: suckling group, W: weaned group.

An LEfSe analysis was performed to assess the changes in cecal taxa with weaning stress. Changes in biomarker content were assessed based on the LDA score (cut of >4), and the phylogenetic distribution of the phyla and genera was computed to identify core taxa (Figure 4A,B). *Campylobacter*, *Clostridiales*, and *Firmicutes* were core taxa in the weaned piglets. In the suckling piglets, *Prevotella_salivae*, *Anctinobacillus_minor*, *Prevotella_bryanti*, *Prevotella_sp_CAG_592*, and *Prevotella_stercorea_CAG_629* dominated. 

**Figure 5:**
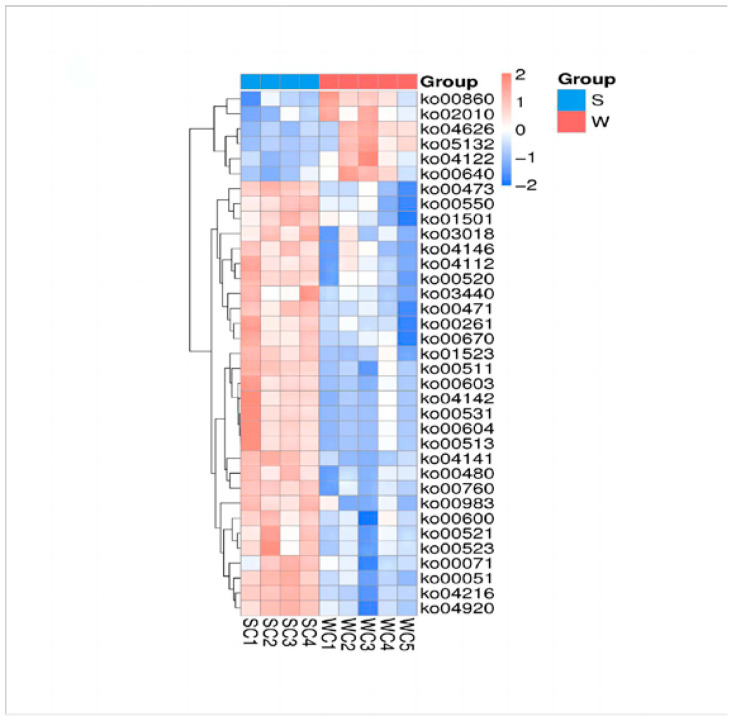
Heatmap of the differential metabolic pathways between S and W piglets.

Using Metastats (Figure 5), we identified the biological pathways associated with the differential taxa. In weaned piglets, porphyrin metabolism (ko00860), propanoate metabolism (ko00640), and salmonella infection (ko05132) were enriched. In suckling piglets, antifolate resistance (ko01532), fatty acid degradation (ko00071), fructose and mannose metabolism (ko00051), amino sugar and nucleotide sugar metabolism (ko00520), and glycine, serine, and threonine metabolism (ko00260) were enriched. These results showed that weaning stress may have adverse effects on the ecological balance and function of the intestinal microbiota.

### 3.4. Metabolic Differences between Weaning and Suckling Piglets

**Figure 6:**
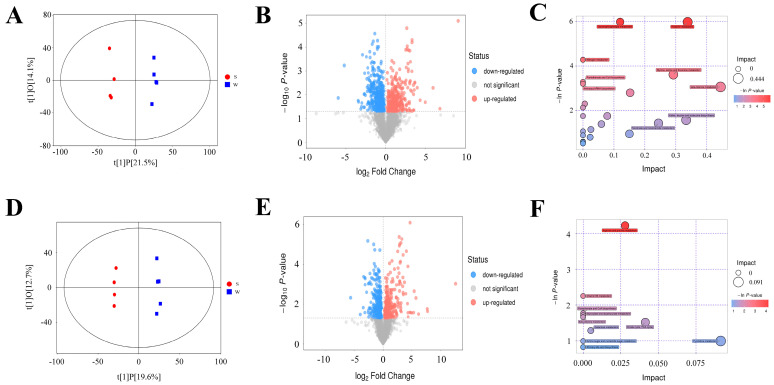
Metabolic analyses of piglets upon weaning stress. (**A**,**D**) Cluster analysis based on the metabolites in positive- and negative-ion modes using the partial least squares discrimination method. Red and blue dots indicate suckling and weaned piglet samples, respectively. (**B**,**E**) Volcano plots of differential metabolites in positive- and negative-ion modes between the S and W groups. Red and blue dots represent significantly upregulated and downregulated metabolites, respectively. (**C**,**F**) Bubble diagram of pathway analysis between weaned piglets and suckling piglets in positive- and negative-ion modes.

We investigated the differences in hepatic metabolite profiles between weaned and suckling piglets using LC−MS/MS in positive- and negative-ion modes. We observed a clear separation in markers between suckling and weaned piglets in both ion modes (Figure 6A,D), suggesting that weaning stress greatly alters metabolic profiles. We identified 757 differential metabolites in the positive-ion mode, with 350 upregulated and 407 downregulated (VIP > 1, FC > 2 or <0.5, and *p* < 0.05; Figure 6B, Supplementary Table S2); 541 differential metabolites were identified in negative-ion mode, with 270 upregulated and 271 downregulated (Figure 6E). We conducted pathway enrichment analysis and observed that the differential metabolites were enriched in glycerophospholipid metabolism, histidine metabolism, glycine, serine and threonine metabolism, and arginine and proline metabolism (Figure 6C,F, Supplementary Table S3).

### 3.5. Integrated Analysis of Metagenomic and Metabolomics Data

**Figure 7:**
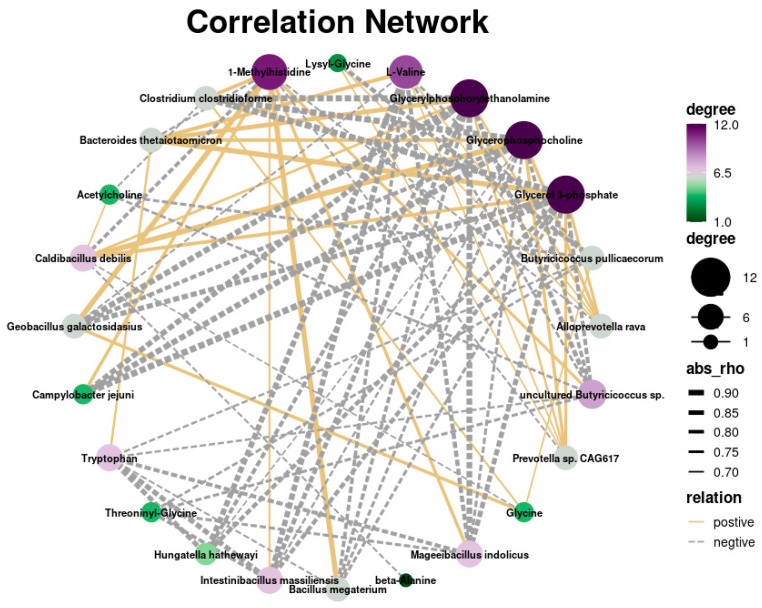
Correlation network analysis between gut microbiota and liver metabolome in piglets.

We conducted an integrated analysis of differentially expressed metabolites and microbiota between the weaned and suckling groups to identify the underlying interactions between gut microbiota and signaling pathways. The results showed that *Campylobacter jejuni* had a positive correlation with histidine-related metabolites and a negative correlation with glycine-related metabolites, whereas Prevotella had a negative correlation with histidine-related metabolites and a positive correlation with glycine-related metabolites (Figure 7).

## 4. Discussion

Weaning stress can cause a variety of adverse reactions in piglets, including diarrhea, reduced growth, intestinal inflammation, and inhibited digestion and absorption [3]. Metabolomic analyses enable the identification of metabolites that modulate diverse biological processes and shape the phenotypes of cells and organisms. In this study, we performed cecal meta-sequencing and liver metabolomic analyses of weaned and suckling piglets to identify differential microbiota and metabolites and their associated biological processes and pathways. Early weaning stress induced damage in the intestinal tissue and mucosa and changed the levels of serum biochemical indicators. The relative abundances of *Bacteroidetes* and *Proteobacteria* decreased and increased, respectively, in W piglets (both *p* < 0.05). The core cecal flora of W piglets were *Campylobacter* and *Clostridium*, while those of S piglets were *Prevotella* and *Lactobacillus*. The differential bacteria were most closely associated with glycerol phospholipid, histidine, nitrogen metabolism, glycine, threonine, serine, β-alanine, and primary bile acid biosynthesis pathways. The differences in glycine–threonine and serine contents may be related to bacterial dysregulation caused by weaning stress. Our integrative analysis of metabolic and meta-sequencing data revealed the main gut microbiota and pathways influenced by weaning stress, providing a critical overview of the biological modules that regulate cellular responses to early weaning stress in piglets.

The gut microbiota of piglets is not yet stable, and a sudden transition from nursing to fiber-rich feed may exacerbate intestinal dysbiosis. Consistent with previous studies on mammals, Firmicutes and Bacteroidetes were found to predominate in the intestinal tract, followed by *Clostridium*, *Proteobacteria*, and *Actinomycetes* [19], which reflects the reliability of the results. Specifically, we observed a significant increase in the abundance of the phyla *Firmicutes* and *Proteobacteria*, while the abundance of *Bacteroidetes* decreased notably in weaned piglets. *Bacteroidetes* play a crucial role in the degradation of metabolic cellulose and hemicellulose [20]. Therefore, the decrease in *Bacteroidetes* and increase in *Proteobacteria* abundance can impact the digestion and absorption of dietary fiber. Prolonged deficiency in dietary fiber can weaken the intestinal barrier, leading to thinning of the intestinal mucus layer and reduced production of SCFAs [21]. *Bacteroidetes* are key members of the cecal microflora in piglets, possessing polysaccharide utilization sites that enable the degradation of cellulose, hemicellulose, starch, β-glucan, and arabinoxylan [22]. *Proteobacteria* are important biomarkers for intestinal epithelial dysfunction. Their β-oxidation-based metabolism of butyrate can cause hypoxia in the colonic epithelium, which affects anaerobic glycolysis in epithelial cells on the intestinal surface, ultimately exacerbating oxidation of the colon epithelium [23]. In our study, the notable increase in the relative abundance of *Proteobacteria* in weaned piglets may be associated with inflammation in the cecum. Overall, these findings suggest the associations between specific microbial phyla and intestinal health and function in weaned piglets.

The core genera in the suckling piglets were *Prevotella* and *Bacteroides*. *Prevotella* is an important SCFA producer in energy metabolism and can utilize xylan, xylose, and carboxymethyl cellulose [24]. Sandberg et al. found that a higher ratio of *Prevotella* to *Bacteroides* was associated with the more favorable metabolism of dietary fiber and glucose in barley, highlighting the importance of microbiota community structure in host metabolism [25]. Moreover, *Prevotella* participates in the regulation of immune responses. Under normal circumstances, the interactions among microbial communities achieve a dynamic balance to control immune responses [26]. However, the dysbiosis of microbial communities can affect mucosal barrier integrity and promote the occurrence of inflammatory diseases. Therefore, integrating the results of this study with previous reports indicated that the gut microbial community of suckling piglets can effectively support the absorption and utilization of xylan, xylose, and carboxymethyl cellulose compared to that in weaned piglets.

We observed a significant increase in the relative abundance of gut bacteria associated with inflammation in the cecum of weaned piglets, such as *C. jejuni*, *Clostridium symbiosum*, and *C. citroniae*. *C. jejuni* is a spiral-shaped bacterium that possesses extracellular capsular polysaccharides. These polysaccharides facilitate its colonization, diffusion, and retention within the intestinal tract, ultimately leading to disease [27]. The bacterium invades and proliferates within intestinal epithelial cells, causing damage, inflammation, and even cell death [28]. Moreover, *C. jejuni* produces an oligosaccharide structure that mimics the GM1 ganglioside present in intestinal epithelial cells. This structure binds to subunits of receptor cholera toxin and *E. coli* heat-sensitive enterotoxin, thereby increasing cell membrane permeability and inhibiting the growth of other bacteria, ultimately disrupting the normal balance of intestinal flora [29]. Our findings suggested that the weaned piglets experienced disruptions in the normal structure of their intestinal flora, allowing the proliferation and colonization of potentially pathogenic bacteria, such as *C. jejuni,* within the intestinal tract. This, in turn, adversely affected the overall physiological health of the piglets.

In this study, *C. jejuni* was more abundant in weaned than in suckling piglets, suggesting a potential relationship between serine metabolism and the improved colonization of *C. jejuni* in weaned piglets. Bile acid metabolism occurs through enterohepatic circulation and is closely related to gut microbiota. Xu et al. suggested that bile acids in the intestine inhibit the growth and colonization of Bacteroidetes while promoting the growth of *Firmicutes* [30]. In this study, the significant reduction in *Bacteroidetes* abundance in weaned piglets may be attributed to the disruption of bile acid metabolism.

Amino acids play a crucial role in various physiological functions, including protein synthesis, immune response, gene expression regulation, and cell signaling [31]. This study revealed that metabolic pathways related to amino acid metabolism, such as serine, glycine, histidine, glycerol phospholipid, threonine, and β-alanine, were significantly enriched in weaned piglets. Among these amino acids, histidine possesses multiple physiological functions, including antioxidative, anti-inflammatory, and immunomodulatory properties. Histidine can inhibit the activation of NLRP3 inflammasome through the SIRT-1-dependent pathway [32]. Moreover, in human intestinal epithelial cells, histidine rescued oxidative stress-related inflammation. The imidazole group of histidine can bind with plasma Zn^2+^ and Cu^2+^, facilitating their intestinal absorption and altering the distribution of metal ions. Zn^2+^ and Cu^2+^ are essential for numerous enzyme activities and normal animal performance [33]. Glycine exerts anti-inflammatory effects by inhibiting inflammatory cytokine activity, such as TNF-α and peroxide secretion, in mouse alveolar macrophages stimulated with LPS. It also suppresses the expression of inflammatory factors, such as IL-6, in the 3T3-L1 cell line and monocytes [34,35,36]. Including 5% glycine in the diet can inhibit the expression of macrophage inflammatory proteins and neutrophil chemokines induced by inflammatory factors, such as TNF-α and IL-1β, in mouse colons, thereby alleviating the occurrence of diarrhea [37]. Serine plays a pivotal role in multiple metabolic processes, such as linking purine synthesis with glycolysis, glutathione and one-carbon metabolism, and pyrimidine, purine, taurine, and glutathione synthesis. Zhou et al. found that adding 0.2% serine to the feed of early-weaned piglets can reduce oxidative stress caused by weaning, thereby improving piglet growth performance and intestinal health [38]. In a porcine intestinal epithelial cell (IPEC-1) model of intestinal inflammation, serine activated the AMPK signaling pathway and effectively maintained intestinal barrier function and permeability [39]. Velayudhan et al. demonstrated that serine promoted the colonization and growth of *Salmonella enterica* in the avian gut [40] (Velayudhan et al. 2004).

*C. jejuni* is associated with diarrhea, gastrointestinal inflammation, and irritable bowel syndrome [41]. Sun et al. demonstrated that orally administering secondary bile acids derived from microbial sources to mice alleviated colitis induced by *C. jejuni*, suggesting that secondary bile acids may inhibit virulence and colonization [42]. We found a significant enrichment of primary bile acid metabolism pathways in the livers of weaned piglets. The negative correlation between *C. jejuni* abundance and glycine contents was likely associated with the covalent binding of glycine to primary bile acids to form secondary bile acids. Although histidine has an anti-inflammatory effect, Gu et al. found that certain pathogens can perceive host-derived nitrate and perform S-nitrosylation on histidine kinase sensor VbrK cysteine 86 to inhibit the expression of inflammatory factors, thereby evading host immune defenses [43]. This potential relationship aligns with the positive correlation observed between *C. jejuni* and histidine metabolites in this study.

It is notable that the sample size used in this study is limited, which may weaken the power of differential metabolites and differential cecal microbiota between the S and W groups of piglets. We will further validate our findings in the current study in a subsequent study with a larger sample size. In addition, differential metabolites and microbiota should be validated by vitro and vivo experiments.

## 5. Conclusions

In conclusion, we showed that weaning stress can adversely affect intestinal physical structure, gut microbiota structure, and metabolic processes, as evidenced by alterations in the microbiome and metabolome of weaned piglets. The dysbiosis in the cecal microflora, reflected in the decreased abundance of *Bacteroidetes* and increased abundance of the potentially pathogenic *C. jejuni*, was accompanied by alterations in gut tissue structure and function. Further integrative analysis revealed that the amino acid and bile acid metabolism pathways were enriched. Further functional analysis revealed that histidine, arginine and proline, glycine, serine–threonine, and bile acid dysregulation may promote dysbiosis and inflammation in weaned piglets. Our study identified key gut microbiota, metabolites, and metabolic processes related to weaning stress, which could inform the development of more effective weaning strategies.

## Data Availability

The metagenomic sequencing data are available under BioProject ID PRJNA553106. The SRA records will be accessible with the following link: https://www.ncbi.nlm.nih.gov/sra/PRJNA999451 (accessed on 17 July 2024).

## Supplementary Materials

The following supporting information can be downloaded at https://www.mdpi.com/article/10.3390/genes15080970/s1, Table S1: Differences of plasma physiological concentrations between weaning and suckling piglets; Table S2: Differential metabolites in positive-ion mode between suckling and weaned groups; Table S3: Pathway analysis of differential metabolites between suckling and weaned groups.
