# Supplementary material for: Integrated Metagenomic and Metabolomics Profiling Reveals Key Gut Microbiota and Metabolites Associated with Weaning Stress in Piglets
Source: Genes (Basel). 2024 Jul 23;15(8):970. doi: 10.3390/genes15080970 (PMC11354067; doi:10.3390/genes15080970)
Supplement: Supplementary file 1 [file genes-15-00970-s001.zip › Table S1.pdf]

Table S1 Differences of plasma physiological concentrations between weaning and suckling piglets

Item	Sucking piglets	Weaning piglets	*P* value
DAO	121.53±25.63	137.49±13.30	0.041
ET	44.06±10.69	57.57±11.71	0.003
Cortisol	120.39±16.34	136.38±23.33	0.038
NE	11.34±1.28	12.57±1.38	0.018
